# Supplementary material for: COVID-19 and the public response: Knowledge, attitude and practice of the public in mitigating the pandemic in Addis Ababa, Ethiopia
Source: PLoS One. 2021 Jan 7;16(1):e0244780. doi: 10.1371/journal.pone.0244780 (PMC7790293; doi:10.1371/journal.pone.0244780)
Supplement: S1 Appendix — (PDF) [file pone.0244780.s001.pdf]

Questionnaire(English Version)

Title of the manuscript:

"COVID-19 and the public response: knowledge, attitude and practice of the public in mitigating the pandemic in Addis Ababa, Ethiopia"

PI: Zlalem Desalegn et al

**Part I: Socio-demographic characteristics**	
Question	Response
1. Age in years	--------------
Sex:	1. Male<br>2. Female
Lives	1. Alone<br>2. With others
Living address	______________________
1. Responsibility within family	1. Head<br>2. Spouse of the head<br>3. Child or relative
Marital status	1. Single<br>2. Married<br>3. Separated/ divorced<br>4. Widowed
Occupation	1. Government employee<br>2. Non-Government employee<br>3. Merchant/ trader<br>4. Other, specify__________________
**Part I: Travel and contact history**	
Have you been traveled in the last three months	1.Yes<br>2.No
Reason for travel	1. Conference<br>2. Visit family<br>3. Business<br>4. Medication

	6. Other, specify__________________
To which COVID-19 affected are have you traveled?	1.China<br>2.Europe<br>3.Middle east/far east<br>4.Other, specify __________________
Did you have a contact witha person who had travel history to COVID-19 affected areas in the last two months?	1. Yes<br>2. No
If you know a person who comes from COVID-19 affected areas, was he/she has signs of any illness that made him/ her take a rest, or seek a medication?	1. Yes<br>2. No
If you know a person who comes from COVID-19 affected areas, was he/she in direct contact with others (in the first two weeks) after arrival to Ethiopia?	1. Yes<br>2. No
Do you remember a family member of a person who came from COVID-19 affected areas, having signs of any illness that made him/ her went to a health facility to seek a medication?	1. Yes<br>2. No
**Part III: Knowledge questions, attitude and practice questions**	
Which of the following do you think are the major signs and symptoms of the disease caused by corona virus?(select all that apply)	1. Fever<br>2. Diarrhea<br>3. Bloody diarrhea<br>4. Bloody sputum<br>5. Swelling of legs<br>6. Cough<br>7. Swelling of mouth/nose<br>8. Red and painful eyes<br>9. Sneezing/runny nose
What are the current ways of prevention of	1. Vaccination

COVID-19?(select all that apply)	2. Anti-viral therapy<br>3. Using masks<br>4. Frequent washing of hands<br>5. Staying at home<br>6. Frequent disinfectant<br>7. Staying >2meters from others
How could a person acquire the Coronavirus disease?(select all that apply)	1. Directly through breathing/ sneezing<br>2. Through a mosquito bite<br>3. Touching mouth and nose through contaminated hand<br>4. Through unprotected sexual intercourse<br>5. Through staying and playing near others<br>6. Not frequently washing while at work<br>7. Using public transport with closed windows<br>8. Opening doors/windows in public places<br>9. Frequent use of disinfectant while at work
Who is at risk of developing the severe form of the corona virus disease? (select all that apply)	1. Diabetic patients<br>2. Hypertensive patients<br>3. People with heart problems<br>4. Pregnant women<br>5. Cancer patients<br>6. Khat chewers/smokers<br>7. Asthmatic patients<br>8. People with COPD
At what age group do you think the coronavirus disease occur?(select all that apply)	1. Children<br>2. Youth<br>3. Elderly
Are children and youth affected by the coronavirus disease?	1. Yes<br>2. No
Is the coronavirus transmittable by shaking/hugging anyone?	1. Yes<br>2. No
Is coronavirus transmittable by mosquito bite?	1. Yes

	2. No
Is the coronavirus transmittable by direct breathing?	1. Yes<br>2. No
Is a person who has a coronavirus detectable by looking at him/ her?	1. Yes<br>2. No
If you got the opportunity, which of the following do you want to apply? (select all that apply)	1.use surgical mask<br>2. Frequently to wash hands with soap and water<br>3. use a hand disinfectant<br>4. Avoid crowd<br>5. Avoid touching eye, nose, and mouth
Do you believe that social/physical distance is necessary to prevent COVID-19?	1. Yes<br>2. No
Do you agree in lockdown is the best strategy to mitigate the pandemic in Ethiopia?	1. Yes<br>2. No
Did you participate in hand washing with soap according to the recommendation(20 second), today?	1. Yes How many times today _____<br>2. No
Did you use sanitizer, today?	1. Yes How many times today _____<br>2. No
Have you stayed for more than 30 minutes, in a room having more than 10 people?	1. Yes<br>2. No
Have you made shaking with anyone today?	1. Yes How many times today _____<br>2. No
Where did you hear about corona-virus, (circle all that apply)and rank them from more-to-low frequent	1.Government owned TV/Radio, facility<br>2.Print media, journals, pamphlets<br>3.Private owned TV/ Radio, facility<br>4.Social Media(Facebook, Twitter, etc)<br>5.Friends, families, relatives
What is the appropriate source of information about COVID-19?(Select all that apply)	1.Government owned TV/Radio, facility<br>2.Private owned TV/ Radio, facility<br>3.Social Media(Facebook, Twitter, etc)<br>4.Print media, journals, pamphlets

	5.Friends, families, relatives
Do you agree that the information provided so far is good enough in protecting from COVID-19?	1. Yes<br>2. No
If you have/see someone with thesign and symptom of COVID-19/pandemic, what do you do?	1.Visiting health institution<br>2.Discussing with relatives<br>3.Using home remedies<br>4.Going to Church/Mosque
