# Supplementary material for: COVID-19 and the public response: Knowledge, attitude and practice of the public in mitigating the pandemic in Addis Ababa, Ethiopia
Source: PLoS One. 2021 Jan 7;16(1):e0244780. doi: 10.1371/journal.pone.0244780 (PMC7790293; doi:10.1371/journal.pone.0244780)
Supplement: S2 Appendix — (PDF) [file pone.0244780.s002.pdf]

ህብረተሰቡ ስለኮቪድ-19 ያለው ዝግጅት፤ግንዛቤና ተግባራዊ ምላሽ የሚዳስስ የምርምር ጥናት

መጠይቅ

መለያ ቁጥር	ጥያቄ	ምላሽ
	**ክፍል አንድ: የማህበራዊ ሁኔq**	
1	ዕድሜ(በዓመት)	--------------------------
2	ጾq	1. ወንድ 2. ሴት
3	የሚኖሩበት ሁኔq	1. ለብቻ 2. ከሌሎች ጋር
4	የሚኖሩበት አድራሻ	--------------------------------------
5	በቤተሰብ ውስጥ ያለዎት ሃላፊነት	1. አባወራ/እማወራ 2. ሚስት 3. ልጅ ወይም ዘመድ
6	የጋብቻ ሁኔq	1. ያላገባ 2. ያገባ 3. የፈ q/† 4. ባል/ሚስት የሞተበት/ባት
7	የስራ ሁኔq	1. የመንግስት 2. የግል 3. ነጋዴ 4. ሌላ ይጠቀስ----
	**ክፍል ሁለት: የጉዞ qሪክ**	
8	ባለፉት ሶስት ወራት ወደውጪ ተጉዘው ያውቃሉ?	1. አዎ 2. አይደለም
9	ለጥያቄ 9 መልስዎ አዎ ከሆነ ፤ለምን ዓላም ጉዞ አደረጉ?	1. ለኮንፈረንስ 2. ለቤተሰብ ጉብኝት 3. ለንግድ 4. ለህክምና 5. ሌላ ይጠቀስ------------------
10	ወደየትኛውም በኮቪድ-19 የተጠቁ ሃገራት ተጉዘዋል?	1. ቻይና 2. አውሮፓ 3. መካከለኛው ምስራቅ/ሩቅ ምስራቅ4. ለሎች ሀገራት ይጠቀስ
11	በኮቪድ-19 ወደተጠቁ ሃገራት ጉዞ ካደረገ ሰው ጋር ግንኙነት ነበሮት?	1. አዎ 2. አይደለም(ወደ ጥያቄ------
12	በኮቪድ-19 ወደተጠቁ ሃገራት ጉዞ ያደረገው ሰው በደንብ የሚያውቁት ከሆነ ከበሽqው የተነሳ እረፍት ወይም ህክምና አድርጎአል?	1. አዎ 2. አይደለም
13	በኮቪድ-19 ወደተጠቁ ሃገራት ጉዞ ያደረገው ሰው በደንብ የሚያውቁት ከሆነ ወደ ኢትዮጵያ ከተመለሰ ጀምሮ ከሌሎች ጋር ቀጥተኛ ግንኙነት ነበረው?	1. አዎ 2. አይደለም
14	በኮቪድ-19 ወደተጠቁ ሃገራት ጉዞ ያደረገው ሰው ቤተሰብ መካከል በደንብ የሚያውቁት ከሆነ ከበሽqው የተነሳ እረፍት ወይም ህክምና ማድረጋቸውን ያውቃሉ?	1. አዎ 2. አይደለም
	**ክፍል ሶስት: ዕውቀት፤አመለካከትና ልምድ የሚዳስሱ ጥያቄዎች**	
15	የኮቪድ-19 በሽqን እንዴት መከላከል እንችላለን?	1. ክትባት 2. ጸረ-ቫይረስ መድሃኒት 3. የፊት ጭምብል ማድረግ 4. በተደጋጋሚ እጅን በመqጠብ 5. እቤት በመቆየት 6. ጀርም ማጽጃዎችን መጠቀም 7. ከማንኛው ሰው ቢያንስ 1 ሜትር መራቅ
16	የኮቪድ-19 በሽq በመጨባበጥና በመተቃቀፍ ይተላለፋል?	1. አዎ 2. አይደለም
17	ቀጥለው ከተዘረዘሩት ውስጥ ምልክቶች የትኞቹ የኮቪድ-19 የሆናል ብለው ያስባሉ?	1. ትኩሳት 2. ተቅማጥ 3. ደም የቀላቀለ ተቅማጥ 4. ደም የቀላቀለ አክq 5. የእግር ማበጥ 6. ማስነጠስና ከአፍንጫ የሚወጣ ፈሳሽ 7. መሳል 8. በአፍንጫና በአፍ ላይ ማበጥ
18	ኮቪድ-19 በትንኝ ይተላለፋል ብለው	1. አዎ 2. አይደለም

	ያስባሉ?	
19	ኮቪድ-19 በቀጥq በመተንፈስ ይተላለፋል ብለው ያስባሉ?	1. አዎ 2. አይደለም
20	በኮቪድ-19 የተጠቃ ሰው ሁሉ ምልክት ያሳያል/በማየት ማወቅ ይቻላል ብለው ያስባሉ?	1. አዎ 2. አይደለም
21	ኮቪድ-19 በህጻናትም የሚከሰት በሽq ነው ብለው ያስባሉ?	1. አዎ 2. አይደለም
22	ኮቪድ-19 በወጣቶችም የሚከሰት በሽq ነው ብለው ያስባሉ?	1. አዎ 2. አይደለም
23	ኮቪድ-19 በማን ላይ የከፋ የጤና እክል/የተወሳሰበ ችግር ያመጣል?	1. ስካር ህመም 2. ደም ብዛት 3. የልብ ህመም 4. እርጉዞች ላይ 5. ህጻናት ላይ 6. በሚቅሙ/በሚያጨሱ/በሚጠጡ ላይ
24	ቀጥለው ከተዘረዘሩት መካከል አንድ ሰው በኮቪድ-19 በሽq ሊያዝባቸው የሚችልባቸው መንገዶች?	1. በማስነጠስና በትንፋሽ 2. በተበከለ እጅ አፍን በመንካት 3. በትንኝ ንክሻ 4. ልቅ በሆነ የግብረ ስጋ ግንኙነት 5. ከሌሎች ጋር በመቀመጥና በመጫወት 6. ከ 1 ሜትር በላይ መራራቅ 7. በተደጋጋሚ በስራ ወቅት እጅን ባለመqጠብ 8. በቤት ቀይq ወቅት እጅን ባለመqጠብ 9. መስኮqቸው በተዘጉ የትራነስፖርት መገልገያዎች መጠቀም 10. ሌላ የቤተሰብ አባል በሽqው ሳይዘው በቤት መቆየት 11. በሆስqፒል አካባቢ ያሉ የበር እጀqዎችን በመጠቀም 12. በስራ አካባቢ በተደጋጋሚ የጸረ-ጀርም ማጽዳዎችን መጠቀም
25	ዕድሉ ቢኖርዎት ከሚከተሉት ውስጥ የትኛውን ይተገብራሉ?	1. የፊት ጭምብል መጠቀም 2. በተደጋጋሚ በውሃና በሳሙና መqጠብ 3. የጸረ-ጀርም ማጽዳዎችን መጠቀም4. ብዙ ሰው በሚሰበሰብበት አካባቢ አለመገኘት 5. አይን፤አፍንጫንና አፍን አለመነካካት
26	በአካላዊ/ማህበራዊ መራራቅ ያምናሉ?	1. አዎ 2. አይደለም
27	በህዝብ/ማህበረሰብ ዘግቶ መቀመጥ ያምናሉ?	1. አዎ 2. አይደለም
28	ዛሬ እጅዎን በተሰጠው መመሪያ መሰረት በሳሙናና በውሃ qጥበዋል	1. አዎ 2. አይደለም<br>አዎ ከሆነ ምን ያህል ጊዜ --------------------------
29	ዛሬ ሳኒqይዘር ተጠቅመዋል	1. አዎ 2. አይደለም
30	ከ 10 ሰወች በላይ በሚገኙበት ክፍል ውስጥ ለ 30 ደቂቃ ቆይተሃል?	1. አዎ 2. አይደለም
31	ዛሬ ከማንኛውም ሰው ጋር ተጨባብጠዋል?	1. አዎ 2. አይደለም<br>አዎ ካሉ ምን ያህል ጊዜ---------------------------
32	ስለ ኮቪድ-19 ከየትኛው የመረጃ ምንጭ ሰሙ?	1. ከመንግስqዊ(ቲቪ፤ራዲዮ...) 2. ከማተሚያ ቤቶች፤ከጋዜጦች፤3. በግል ከተያዙ ቴሌቭዥን ድርጅቶች፤ራዲዮ ጣቢያዎች4. ከማህበራዊ ድረ-ገጾች 5. ከቤተሰብ፤ከጋደኛ፤ከዘመድ
33	ከተዘረዘሩት የመረጃ መንጮች የትኛ ውን በይበልጥ ያምናሉ?	1. ከመንግስqዊ(ቲቪ፤ራዲዮ...) 2. ከማተሚያ ቤቶች፤ከጋዜጦች፤3. በግል ከተያዙ ቴሌቭዥን ድርጅቶች፤ራዲዮ ጣቢያዎች4. ከማህበራዊ ድረ-ገጾች 5. ከቤተሰብ፤ከጋደኛ፤ከዘመድ
34	የተሰጥዎት መረጃ የኮቪድ-19 በሽqን ለመከላከል በቂ ነው ብለው ያምናሉ?	1. አዎ 2. አይደለም
35	የኮቪድ-19 በሽq ምልክቶችን በራስዎ ወይም በሌሎች ላይ ቢያዩ ምን	1. ወደ ጤና ተቀም ለምርመራ መሄድ<br>2. ከሌሎች ጋር ስለጉዳዩ መወያየት 3. የቤት
