# Supplementary material for: COVID-19 and the public response: Knowledge, attitude and practice of the public in mitigating the pandemic in Addis Ababa, Ethiopia
Source: PLoS One. 2021 Jan 7;16(1):e0244780. doi: 10.1371/journal.pone.0244780 (PMC7790293; doi:10.1371/journal.pone.0244780)
Supplement: Attachment — Submitted filename: Response to Reviewers.docx [file pone.0244780.s005.docx]

**Point by point response for Editor and reviewers comment**

**Manuscript number**: PONE-D-20-17538

**Manuscript title: "**COVID-19 and the public response: knowledge, attitude and practice of the public in mitigating the pandemic in Addis Ababa, Ethiopia"

**Dear editor and the reviewer,**

First of all, we would like to thank the journal team members, the journal editor and the respective reviewers for taking their time and sending valuable suggestions for our manuscript.

As per the request, we incorporated all the relevant changes to the revised manuscript with track change. Additionally, a detail point by point response for each editors and reviewers comment enclosed with this letter.

**With kind regards,**

Zelalem Desalegn Woldesonbet

PI and corresponding/submitting author

Assistant professor, Department of Microbiology, Immunology and Parasitology

School of Medicine, College of Health Sciences

Addis Ababa University

**Response to reviewers and editor comments:**

**Reviewer #1:** 
COVID-19 and the public response: knowledge, attitude and practice of the public in mitigating the pandemic in Addis Ababa, Ethiopia
The authors have made their efforts and the manuscript becomes far better than before. However, the manuscript still could be strengthened especially in the discussion.
1. LINE 166 – I think it is a typo. “839 84 per site)”.

Thank you for your thoughtful comment. We appreciate the comment and corrected in the revised version **( Please refer line number 166 of the revised version)**
2. LINE 232 – 36.7 % is not the large majority. You can simply say” government employee and non-government employee occupied one third each (36.7% and 34.7%) followed by …”.

Well taken. We accept the comment and we presented the data in the way you have recommended **( Please refer line number 232-236 of the revised manuscript)**
2. Table 1 - Remove the unknown row if it does not exit (0%) or remain if occupied some %

Well taken. We have removed the unknown row ( **Please refer table 1)**
3. Some parts of table 4 are missing. I think it is a formatting error.

Well taken. We included the missed p-value **( Please refer table 4)**

4. Table 5 - I accept author explanation. It is better to present as a correlation matrix table.

We appreciate your positive feedback.
5. Table 8 - does not make 100% in cumulative. Please check.

Well taken. Yes, as you have sated it has to give 100%; however, the reason for not making 100% is due to no response for some of specific questions in the checklist **( please refer table 8)**

6. Discussion – still have lots of room for improvement for better, concise and comprehensive. Repeating the finding statements and sentences from the methods (e.g. LINE 341, 369, 377, etc.) should be avoided here. Information is repeated in many places (e.g. LINE 329 vs 360 vs 402). There are ways to discuss better without repeating findings and methods. The discussion part includes mostly comparison while missing potential reasons why the current results were found.

We appreciate your comments. Accordingly, we removed the finding statements and sentence directly taken from result section. We also removed information repeated and tried to discuss better without repeating findings and methods **(Please refer the whole discussion part for better clarification).**

**Reviewer #2:**

 I would like to thank the authors for sufficiently addressing all the comments raised by the reviewer. Well done.

Additional Editor Comments (if provided):
1. Still needs to do English language editing by the native speaker or the recognized English language editing service and also needs to improve the discussion part up to standard.

Well take. We appreciate the comment forwarded by editor for the betterment of the revised manuscript. We have gone through the manuscript thoroughly to improve the language and present the research data fulfilling the journal requirements.

Additionally, the manuscript has been sent out for recognized language editing service.

The authors strived to their best to make the discussion part very comprehensive, concise and clear to improve it up to the standard.
