# Supplementary material for: COVID-19 and the public response: Knowledge, attitude and practice of the public in mitigating the pandemic in Addis Ababa, Ethiopia
Source: PLoS One. 2021 Jan 7;16(1):e0244780. doi: 10.1371/journal.pone.0244780 (PMC7790293; doi:10.1371/journal.pone.0244780)
Supplement: Attachment — Submitted filename: Reviewer comments.docx [file pone.0244780.s004.docx]

**PONE-D-20-17538-R1**

**COVID-19 and the public response: knowledge, attitude and practice of the public in mitigating the pandemic in Addis Ababa, Ethiopia**

The authors have made their efforts and the manuscript becomes far better than before. However, the manuscript still could be strengthened especially in the discussion.

- LINE 166 – I think it is a typo. “839 84 per site)”.
- LINE 232 – 36.7 % is not the large majority. You can simply say” government employee and non-government employee occupied one third each (36.7% and 34.7%) followed by …”.
- Table 1 - Remove the unknown row if it does not exit (0%) or remain if occupied some %
- Some parts of table 4 are missing. I think it is a formatting error.
- Table 5 - I accept author explanation. It is better to present as a correlation matrix table.
- Table 8 - does not make 100% in cumulative. Please check.
- Discussion – still have lots of room for improvement for better, concise and comprehensive. Repeating the finding statements and sentences from the methods (e.g. LINE 341, 369, 377, etc.) should be avoided here. Information is repeated in many places (e.g. LINE 329 vs 360 vs 402). There are ways to discuss better without repeating findings and methods. The discussion part includes mostly comparison while missing potential reasons why the current results were found.
